# Using Responsive Feedback in Scaling a Gender Norms-Shifting Adolescent Sexual and Reproductive Health Intervention in the Democratic Republic of Congo

**DOI:** 10.9745/GHSP-D-22-00208

**Published:** 2023-12-18

**Authors:** Kathryn M. Barker, Jennifer Gayles, Mariam Diakité, Florentine Gracia Diantisa, Rebecka Lundgren

**Affiliations:** a Center on Gender Equity and Health, University of California San Diego, La Jolla, CA, USA.; b Department of Global Health, Save the Children Federation, Washington, DC, USA.; c Independent consultant, Bamako, Mali.; d Save the Children International, Kinshasa, Democratic Republic of the Congo.

## Abstract

**Program Description:**

Growing Up GREAT! (GUG) is a sexual and reproductive health (SRH) program for adolescents aged 10–14 years in Kinshasa, Democratic Republic of the Congo (DRC). The multilevel program takes an ecological approach to foster community examination of gender inequitable norms and to increase adolescents' SRH knowledge, skills, and gender-equitable attitudes. GUG design, piloting, and scale-up were informed by a theory of change and responsive feedback mechanisms (RFMs) during piloting and scale-up.

**Responsive Feedback Mechanisms:**

The program engaged stakeholders via quarterly learning meetings to review monitoring data, evaluation results, and practice-based knowledge and to subsequently identify challenges and develop solutions. The program commissioned rapid research on specific intervention elements to improve implementation and documented scale-up learnings using the World Health Organization/ExpandNet framework.

**Achievements:**

RFMs used in the pilot period allowed the program to address community concerns by intensifying orientation activities with parents and schools, shifting the calendar of activities to increase male engagement, and increasing facilitator training length to improve facilitation quality. Using RFMs during scale-up prompted further adaptations for program sustainability, including recommendations for task-shifting from NGO facilitators to community health workers.

**Conclusion:**

GUG used RFMs from pilot through scale-up to foster a learning culture among local partners, implementers at headquarters, and global research partners. Using responsive feedback (RF) enabled timely response to the evolving implementation context, resulting in strategic program adaptations that fostered increased community support of the project. Other successes due, at least in part, to this RF approach include incorporation of the program into DRC's national adolescent health strategy, and rapid response to the COVID-19 pandemic in educational strategies for program beneficiaries.

## Abstract (precis)

Responsive feedback mechanisms—especially a culture of learning among donors, researchers, and implementers—facilitated timely adaptations and scale-up efforts of an adolescent sexual and reproductive health program in the Democratic Republic of the Congo.

## Teaser

**Key Findings:**

- The use of responsive feedback mechanisms (RFMs) and a theory of change enabled necessary program adaptations during piloting and scale-up of the Growing Up GREAT! project.
- These adaptations allowed the program to respond in meaningful ways to local communities, leading to improvements in implementation and in response to system shocks such as election-related instability and COVID-19–related school closures.
- Donors, researchers, and implementing institutions fostered a culture of learning at all levels in the programmatic ecosystem to ensure that those most knowledgeable about needed course corrections were able to voice them.

**Key Implication:**

- RFMs should be prioritized for course corrections to strengthen program sustainability.

## INTRODUCTION

Public health interventions are designed and implemented in complex and dynamic systems across cultures and time. A responsive and responsible implementation strategy will seek to maximize improvements in the lives of people for whom the interventions are designed. However, dominant models of global health intervention programs do not account or allow for the complexity and unpredictability inherent in implementation in dynamic environments.^1^ This is in part due to donor-driven metrics and timelines, an overreliance on randomized control trials as the gold standard for evaluation, and the weakness of clinical empirical models to assess social change efforts.^2^ In recent decades, numerous thought leaders and organizations have recognized the limitations of the standard development paradigm and have called for new strategies that engage global and local stakeholders via timely data use and other learning strategies to systematically adapt and improve programs.^1^^,^^3^^,^^4^ Stemming from these emerging perspectives, a number of complementary approaches have been established to guide implementers in how best to respond and adapt to complexity.

One such approach, responsive feedback (RF), is a systematic process for linking ongoing implementation learnings to modifications in project design that promotes reflection and discussion between project designers, implementers, researchers, and decision-makers to provide timely assessments and actionable feedback, thereby enabling programmatic course corrections needed to achieve intended outcomes and maximize health improvements.^1^ Another similar framework known as Collaboration, Learning, and Adapting calls for using a set of tools developed by the U.S. Agency for International Development and their implementation partners to aid in learning and adapting throughout the project life cycle.^3^ Common elements to the RF and Collaboration, Learning, and Adapting frameworks include the following program characteristics: (1) an agile and flexible design; (2) adaptive to context and situation; (3) openness to test and change; (4) inclusive of and responsive to needs of implementers and decision-makers; (5) use of actionable data.^1^ The capacity and capability to gather evidence to fill gaps in knowledge is foundational to the RF approach.^5^ The learning agenda that guides evidence generation is set by a program's theory of change (TOC), which helps program implementers and researchers prioritize “what” to observe and “when” to observe by explicitly documenting assumptions in cause-effect relationships between activities and outcomes.^1^^,^^6^ This allows implementing staff, researchers, and others involved in the program to critically appraise these assumptions, with support from responsive feedback mechanisms (RFMs)—tools that support the learning process and adaptive thinking.^1^

Despite growing recognition of the value of using RF loops to enable course corrections, published evidence documenting the benefits and strategies used to successfully apply RFMs is scant. Viswanath et al. discuss the multiple challenges associated with applying RFMs, including organizational structure and culture, insufficient resources, and lack of capacity.^1^ However, they also assert that these barriers can be overcome with sufficient stakeholder commitment. Use of feedback during the design phase of a pilot to develop a digital service business for Nigerian patent and proprietary medicine vendors is explained by Wright et al.'s article highlighting the importance of a TOC to identify pathways and markers of course correction.^7^

Despite growing recognition of the value of using RF loops to enable course corrections, evidence documenting the benefits and strategies used to successfully apply RFMs is scant.

The use of RF is also critical to the successful scale-up of proven interventions. Although many definitions of scale-up exist, the World Health Organization/ExpandNet Consortium describes scale-up as “deliberate efforts to increase the impact of…innovations successfully tested in pilot or experimental projects so as to benefit more people and to foster policy and program development on a lasting basis.”^8^ To successfully scale a proven pilot program, several requirements must be met. First, scale-up depends on adaptation, as it is impossible to reproduce any activity or program without accommodating the contexts to which it will be spread or in which it will be institutionalized.^9^ This adaptation must occur while retaining fidelity to outcomes and “core” program mechanisms. However, the process for achieving these outcomes may change. A second requirement is that of adaptive management—a style of activity management that emphasizes continuous collection of information to flag needed improvements and facilitate adaptation.^10^ The data collection and monitoring approaches needed for scale-up must provide information that yields a real-time dynamic picture of progress to guide efforts.^11^ During the scale-up period, the focus is on measuring processes to ensure the innovation is implemented with fidelity at an acceptable pace and achieves desired coverage.^12^ A final requirement is an awareness of complexity—at the contextual, temporal, and interpretive levels—to consider how the environment and implementation process shape intervention outcomes; how interventions evolve over time due to new understandings, constraints, opportunities, and priorities; and the many viewpoints and perspectives held by the various stakeholders.^13^ Each of these requirements—adaptation, adaptive management, and complexity awareness—necessitates the application of RF approaches.

We document the RF approaches applied from piloting through scale-up and the corresponding results from the Growing Up GREAT! (GUG) intervention (known locally as Bien Grandir!) in Kinshasa, Democratic Republic of the Congo (DRC). In this article, we describe the RFMs used to adapt, pilot, and scale this gender norms–shifting intervention for very young adolescents (VYAs) aged 10–14 years over 6 years; discuss results of the application of RFMs to implementation; and draw conclusions about the value of applying RF to program adaptation and scale-up efforts.

## GROWING UP GREAT! INTERVENTION DESCRIPTION

**FIGURE 1:**
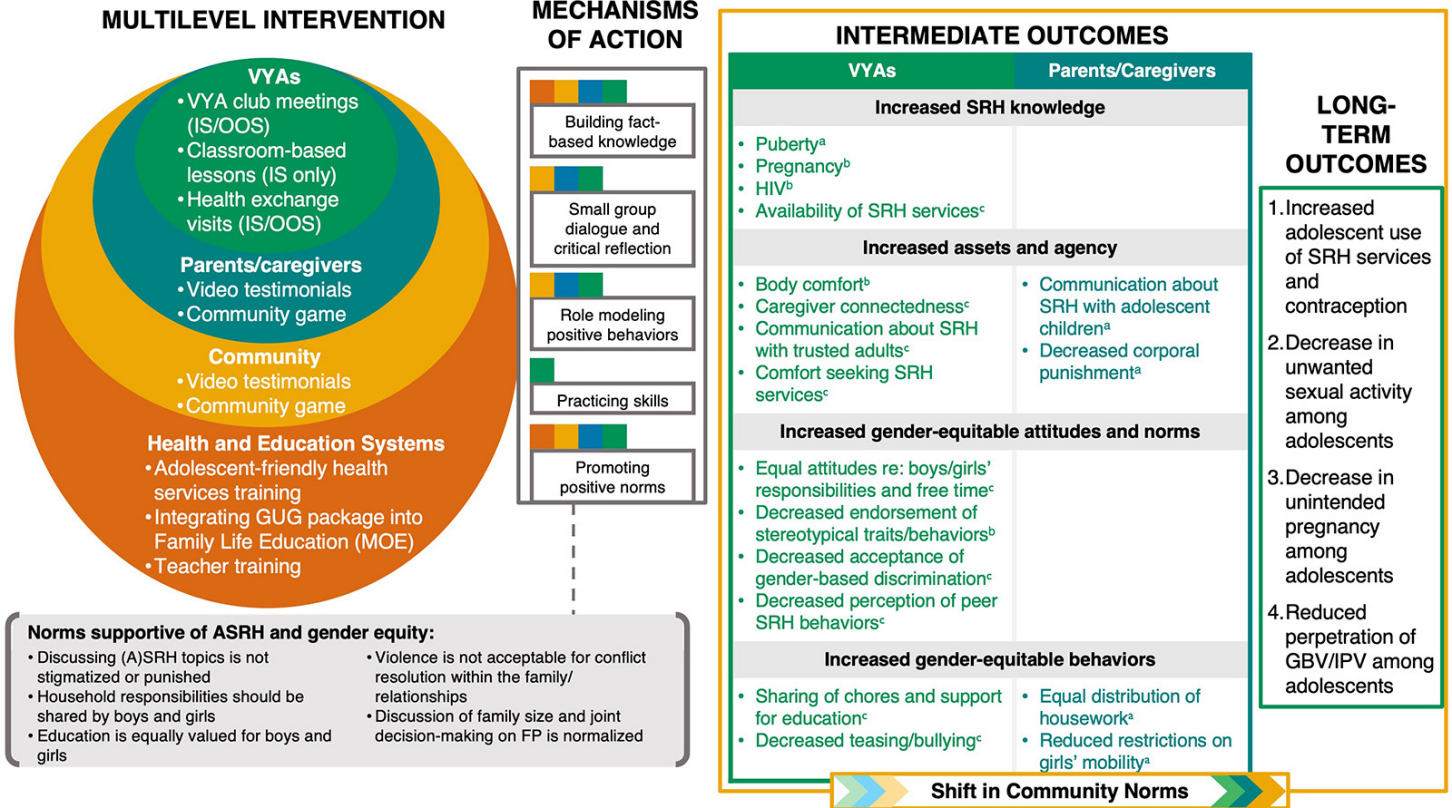
Growing Up GREAT! Theory of Change Abbreviations: ASRH, adolescent sexual and reproductive health; FP, family planning; GBV, gender-based violence; GEAS, Global Early Adolescent Study; GUG, Growing Up GREAT!; IPV, intimate partner violence; IS, in-school; MOE, Ministry of Education; OOS, out-of-school; SRH, sexual and reproductive health; VYA, very young adolescents. ^a^ Outcomes not measured by the GEAS. ^b^ Outcomes fully measured by the GEAS. ^c^ Outcomes partially measured by the GEAS.

A social norms exploration conducted with local staff and stakeholders identified a related set of inequitable norms linked to girls' education, chore burden, and free time at home; access to adolescent sexual and reproductive health (ASRH) services; and violence. GUG was designed as a scalable gender norms–shifting^14^ approach to foster a critical examination of these local gender norms, increase ASRH knowledge and assets, and cultivate gender-equitable and nonviolent attitudes and behaviors among VYAs. From September 2017 to May 2018, Save the Children, in partnership with 8 local community-based organizations (CBOs), implemented the multilevel intervention by engaging VYAs, their caregivers, communities, teachers, and health providers (Figure 1). More than 2,300 VYAs participated in school and community-based adolescent groups that used a toolkit with interactive, age-appropriate materials to learn about ASRH and explore related social norms during weekly mixed-sex group sessions. More than 2,000 parents and caregivers participated in 6 hour-long reflective group discussions prompted by testimonial videos featuring positive norms-driven behaviors. Additionally, more than 2,200 community members, including traditional and religious leaders, joined discussions about ASRH and norms as part of the community game during community-wide sessions. At the education systems level, teachers received training to integrate GUG materials into the national Family Life Education (FLE) curriculum; this reinforced information for school club members but also reached VYAs who were not members of school clubs. Importantly, it also provided a path to scale-up and sustainability within government systems. At the health systems level, facility-based health providers received training to provide adolescent-friendly services and hosted facility visits for VYAs.

Since the beginning of the 7-year project life cycle (2016–2023), GUG has also engaged researchers, scale-up experts, and implementation scientists to (1) build the evidence base on VYA-specific outcomes, including the formation of gendered attitudes via longitudinal data from a 5-year cohort study as part of the larger Global Early Adolescent Study^15^^,^^16^; and (2) institutionalize locally prioritized intervention elements within Congolese policies, systems, and structures. Over the final 3 years of the project life cycle, scale-up efforts accelerated, resulting in vertical scale-up achievements, such as integrating GUG into the FLE in-service training for teachers and including GUG programmatic elements into the Ministry of Health (MOH)'s National Program for Adolescent Health (Programme National de Santé de l'Adolescent) 5-year strategic plan (2021–2025).^17^ The project is described in greater depth elsewhere.^18^

## ENABLING CONDITIONS FOR RESPONSIVE FEEDBACK

From project inception, the GUG team purposefully established values, systems, and processes to support a learning culture. This culture was grounded in systems thinking, which recognized the complex, interconnected social and institutional factors that influenced project activities and outcomes.

From project inception, the GUG team purposefully established values, systems, and processes to support a learning culture.

### Stakeholder Engagement

The project engaged a wide range of stakeholders, including representatives from the Ministry of Education (MOE) and MOH, community-based volunteers, parents/caregivers, and the youth themselves. All stakeholders had a strong working knowledge of the local context and cultures at play within intervention sites. Efforts were made to involve these individuals in every phase of the project, starting with intervention design and continuing through to pilot and scale-up. In 2016, a multidisciplinary Stakeholder Reference Group (SRG) was formed to serve as GUG's technical advisory committee, cochaired by the National Program for Adolescent Health (MOH/Programme National de Santé de l'Adolescent) and the Department of Family Life Education (MOE/Direction de l'Education à la Vie Courant). Comprised of nearly 50 members from governmental and civil society organizations who were engaged in ASRH, the SRG was the primary body responsible for promoting program scale-up, institutionalizing the intervention in ministry policies and planning documents, and strengthening the capacity of local CBOs versed in ASRH to take up GUG approaches. The SRG achieved this by providing technical input, sharing information and tools across government structures and the wider development community, mobilizing resources, and advocating within government agencies for continued investments in VYAs. The Ministry of Gender, Family and Children; the Ministry of Social Affairs; and the Ministry of Youth and Sports also coordinated with project actors via their active participation in the SRG.

In addition, starting in 2017, a Youth Advisory Council comprising in-school and out-of-school VYAs participating in GUG, alongside members of the Kinshasa Youth Parliament, provided feedback on project implementation and results from a community and youth-based perspective and helped the consortium ensure accountability to primary beneficiaries (i.e., VYAs). Youth also led a qualitative participatory evaluation that engaged VYAs as researchers to identify the most important changes resulting from the intervention in their families, schools, and communities.

### Learning Culture

Over the life of the project, implementing partners were included as equal partners in program implementation, monitoring, and learning. The core team, composed of staff from Save the Children and researchers from the Institute for Reproductive Health at Georgetown University, worked to strengthen leadership capacity among local staff and partners through training in the program, as well as the theory and application of norms-shifting interventions. This built staff and partner confidence to share observations gained during implementation. These observations (often called practice-based learning)^19^ were given the same weight as monitoring data or rapid study results. Most importantly, reflection and continuous learning were institutionalized via learning meetings that brought together all implementing actors.

### Theory of Change

A TOC developed early in the project guided implementation and articulated assumptions behind how the multilevel intervention was assumed to lead to positive change in key ASRH behaviors. The TOC drew on emerging social norms theory that identifies key attributes of norms-shifting interventions^20^ and built on the global TOC of the U.S. Agency for International Development–funded Passages Project,^21^ which highlights several broad, interconnected implementation strategies that operationalize these attributes. The GUG TOC was developed simultaneously with the intervention and continuously reviewed against learnings from implementation and evaluation. Implementers and researchers revised it substantially in 2018 after some months of implementation to more explicitly map intervention activities to key outcomes. It underwent another major revision in 2020 to reflect lessons learned from piloting and scale-up and was shared with government and CBO partners (Figure 1). The first iteration depicted change within the family and community combining to create a more supportive environment, in turn, driving change among adolescents. Additionally, anticipated outcomes were very tied to the social norms targeted by GUG. Subsequent versions of the TOC emphasized the influence of multiple activities at all levels of the socioecological model and the specific mechanisms of change at each level. They also integrated program learning and evaluation data to refine outcomes that more realistically reflected what the intervention could achieve within the project timeline. Notably, the final TOC is the only version to depict how attitudes, behaviors, and norms all change and influence one another simultaneously.

### Time Frame and Donor Support

Fortunately, the donors supporting this endeavor, the U.S. Agency for International Development and the Bill and Melinda Gates Foundation, committed adequate resources over a 6-year period to allow for the establishment of an effective learning team and a long enough time frame in which to deploy program adjustments prompted by RF. This was particularly important given the challenges in DRC during this time, which encompassed civil unrest, elections, and the COVID-19 pandemic.

## RESPONSIVE FEEDBACK MECHANISMS

Building on stakeholder engagement, a learning culture, and the TOC, the GUG team applied several RFMs during the project life cycle. We used simple and coordinated monitoring tools, developed a learning agenda, developed a learning matrix to document lessons learned, and commissioned rapid research on specific intervention elements. During scale-up, we adapted the World Health Organization/ExpandNet's Implementation Mapping Tool (IMT)^22^ to support and document learning and adaptation related to institutionalization.

**FIGURE 2:**
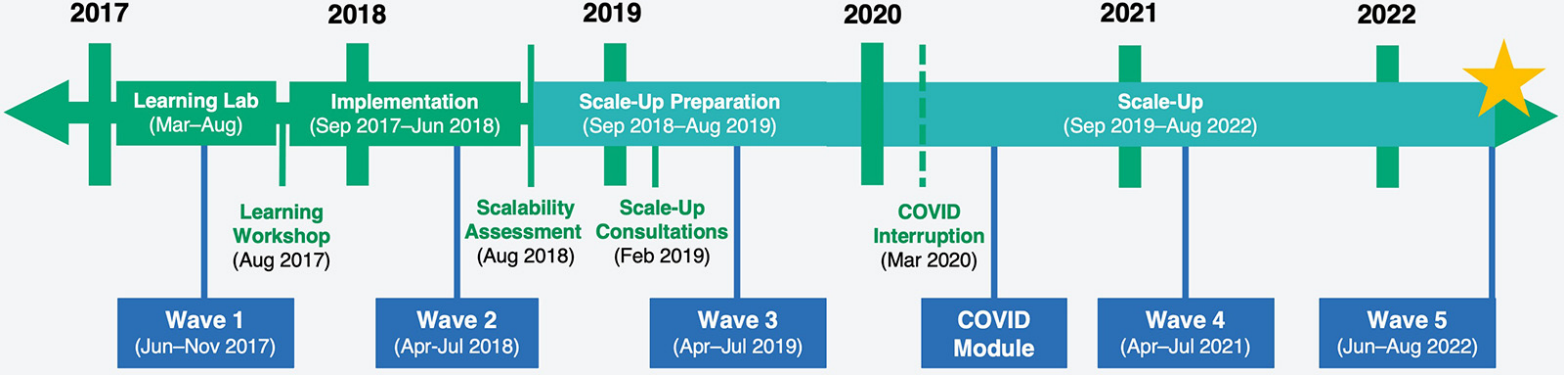
Timeline of Growing Up GREAT! Implementation

RFMs were operationalized in each phase of the project life cycle to maximize opportunities for learning (Figure 2). Phase 1 of the program (2016–2017) included rapid formative research, drawing on workshops with youth, parents, and Ministry stakeholders to adapt a previously validated intervention (albeit from a rural Ugandan context) to urban Kinshasa. Phase 2 (March–August 2017) was known as the learning lab, in which a rapid pilot test occurred to determine how to adjust the intervention before implementation. During this 6-month phase, regular activity monitoring began, and learning meetings were initiated. These meetings brought together implementers to review available data, discuss observations, and recommend adaptations and improvements as necessary. Phase 3 (2017–2018) was the official pilot, conducted in 2 communities (Masina and Kimbanseke) and assessed by a pre- and post-test control group design outcome evaluation as part of the Global Early Adolescent Study.^15^ Project monitoring and documentation efforts that started during the learning lab (Phase 2) were strengthened and continued during this phase. Implementation learning and evaluation findings pointed to a handful of needed revisions to the intervention, especially the video discussion guides for caregiver and community sessions. The guides were revised to include more content on open and respectful communication with VYAs and nonviolent discipline, a topic frequently requested by participants during implementation. Phase 4—preparing to scale—began at project inception and was informed by all other phases. A preliminary scale-up plan was developed during a workshop in August 2016 in Kinshasa. This plan focused on generating credible, actionable evidence; engaging stakeholders; and developing a scalable intervention. This and subsequent workshops deepened scale-up understanding and planning and built the project team's ability to troubleshoot problems as they arose. After the pilot (Phase 3), the team held a workshop to review evaluation results and discuss implementation experience, informed in part by monitoring data and documentation. The goal of these data-informed discussions was to decide whether the results justified further implementation and scale and to determine whether the intervention met the CORRECT (Credible, Observable, Relevant, Relative advantage, Easy to install, Compatible, Testable) criteria for scale-up.^23^ The decision was made to continue to the scale-up phase, although learnings from the workshop prompted a number of changes to the intervention to ensure maximum potential for integration and sustainability. Thus, the implementation learning was important not only for adjusting the intervention during Phase 3 (pilot) but also for informing adaptation before planned scale-up, which began in 2019.

RFMs were operationalized in each phase of the project life cycle to maximize opportunities for learning.

### Monitoring Tools

Save the Children developed monitoring tools to collect data on the coverage, pace, and quality of program implementation. These included forms to track participation and use of materials by VYAs and parent and community groups, as well as qualitative tools to document challenges, lessons, and norms-shifting mechanisms. Qualitative data allowed the team to track diffusion of ideas throughout the community, monitor and address pushback, and better understand social change pathways. Quality benchmarks were added based on the learning lab (Phase 2). These were collected during supervision visits to evaluate fidelity of the intervention, that is, the extent to which activities were implemented as intended. Quality benchmarks were especially helpful in identifying facilitator capacity needs during supportive supervision visits. In addition, staff from local CBOs who helped implement GUG noted challenges and learnings in their monthly reports. The Save the Children team in DRC held internal monthly reflection meetings to review monitoring data, tools, processes, and communication chains, as well as to discuss challenges. Data were also reviewed during multistakeholder learning meetings, during which implementing partners discussed and proposed solutions (adaptations) for improved implementation.

### Learning Agenda

We established quarterly learning meetings (“pause and reflect sessions”) during implementation (Phase 2–3) that brought together all program actors—from direct implementers (e.g., teachers and health providers) to local partners (e.g., CBOs, MOE, and MOH) to technical experts from the government. However, busy schedules among ministry partners and the common practice of appointing a colleague to attend in one's stead meant that meetings often started late and sometimes benefited from less informed reflection. The purpose of learning meetings was to report on activities; review and interpret monitoring and supervision data; cultivate critical reflection and discussion with implementing partners and stakeholders on key challenges, successes, and lessons learned; and agree upon any needed adjustments.

The learning meetings aimed to report on activities, review monitoring data, and cultivate critical reflection and discussion with stakeholders on challenges, successes, and lessons learned.

### Learning Matrix

Discussion and decisions were documented in a learning matrix, a table that tracked challenges, successes, lessons learned, and proposed adaptations for each GUG component/activity. During scale-up (Phase 5), we replaced the learning matrix with the IMT, a validated tool developed by ExpandNet to document challenges and successes encountered during the process of institutionalizing GUG within government platforms. We also documented all proposed and implemented program adjustments and their results related to program quality and/or outcomes.

### Rapid Learning Studies

Toward the end of the implementation period (Phase 3), 3 small qualitative investigations allowed us to examine intervention components that the Save the Children team identified as needing further attention, notably those that were not included in the intervention's formal evaluation. These intervention components included parent/caregiver sessions, teacher-led integration of GUG materials into the national FLE curriculum and support for school-based clubs, and health exchange visits. The first study examined the feasibility and effectiveness of the parent/caregiver sessions and provided critical information about who participated, what caregivers took away from the sessions, and how to improve the sessions' effectiveness. The second study, with teachers and school leadership, provided a deeper understanding of the frequency and quality of activities in school clubs versus classroom lessons and furnished information on how teachers used materials to complement the national FLE curriculum. The third study examined the feasibility, utility, and potential for scale of GUG's health system–level activities.

## RESULTANT PROGRAMMATIC ADAPTATIONS

Development of a learning culture and application of RFMs resulted in concrete program adjustments that enhanced the effectiveness and scalability of GUG. These changes included increased program acceptability within the community and intervention sites; expansion to include engagement of male caregivers; revisions to implementation materials; improved facilitation quality; and the ability to respond rapidly to changes in the environment.

### Understanding and Addressing Community Pushback

Implementing partners initially encountered resistance from caregivers to children participating in VYA clubs, a problem common to ASRH programs globally. Schools had leveraged parent-teacher associations and regular school communication channels to inform caregivers about GUG, and although all had signed consent forms, some stopped their children from participating when they saw take-home materials. By reviewing qualitative monitoring data and having discussions with CBO staff, it became clear that resistance was mostly because of a lack of awareness of GUG's focus on ASRH among parents of GUG's purpose. Although some parents disapproved of discussing ASRH generally, others were surprised to see materials and supported their child's participation once they understood the program's purpose. Understanding the cause of the pushback made it easy to propose an appropriate solution. We adjusted the timing of the first parent video session to hold it before VYA club sessions began. We used the session to orient parents to the program aims and content so they were not surprised when children brought home materials to share and discuss.

### Understanding and Addressing Pushback in Private Schools

GUG worked with all 3 types of schools in Kinshasa—public, parochial, and private. Private school teachers and administrators were enthusiastic about taking up the intervention. However, owners of the private schools—who have unique decision-making power about curriculum and school management—were fearful that parent and community disapproval would affect enrollment at their schools. Local partners noted challenging conversations with several of these individuals and also raised the issue during our first learning meeting. The ensuing discussion revealed that this was a common problem in many private schools. A simple solution was proposed. Because of the efforts invested in establishing a strong partnership with the MOE throughout the project life cycle, we were able to engage officials who oversaw private schools to advocate to owners the importance of the program and gain their support. In a majority of cases, this approach was successful in convincing school owners to implement GUG.

### Engaging Male Caregivers

Early monitoring data showed weak engagement of men—specifically male caregivers—in the intervention activities designed for them. For instance, only 20% of participants in the video sessions with parents were men. This weak engagement was confirmed by implementer observation captured in learning documentation and meetings. Additionally, we heard that a small number of participating female caregivers discussed program content with their spouses or peers. While we were aware that there were multiple factors influencing men's participation, we made a few simple adaptations that were feasible from a program perspective. First, we experimented with different days and times for caregiver sessions to see if we could better accommodate men's schedules. Despite significant efforts to make program activities more convenient and attractive to male caregivers, these strategies were ultimately not successful. With this information, we pivoted again, attempting to reach men indirectly, but systematically through organized diffusion; we encouraged all participants (again, mostly women) to share what they learned with their spouses and other members of their households. Diffusion was not tracked because the program evaluation was designed to assess change at the adolescent rather than family level.

Efforts to increase direct male engagement in program activities were unsuccessful, so the program pivoted to reach them indirectly.

### Revision of Intervention Materials

Results of the learning studies signaled the need to revise the intervention to increase caregiver comfort and confidence with raising and discussing sensitive topics such as puberty and adolescent sexuality—topics that are traditionally seen as uncomfortable or taboo topics for parents to discuss with children. The video discussion guide was expanded from 1 to 6 guides (1 per video) to provide additional facilitator guidance and discussion prompts. This improved usability and flow and increased discussion of positive behaviors among parents. The revised guides also provided more background information, including a description of the group-based dialogue approach and objectives and recommendations for identifying locations conducive to the caregiver and community sessions.

### Improved Facilitation Quality

Program quality indicators revealed limitations in the facilitation of activities, especially in peer-facilitated school clubs. This was associated with reduced quality and depth of participant engagement and discussion and was the subject of animated discussion at several learning meetings. Monitoring data also revealed much higher participation in the community-based sessions facilitated by CBO animators (81%) as compared to the school-based peer-facilitated sessions (39%). Given the importance of quality facilitation in fostering critical reflection and discussion, we revised both the training and supervision strategies of the facilitators. We extended training by 1 to 2 days (depending on the type of facilitator) and worked to ensure that teacher focal points were providing adequate support for peer-led school clubs. We also increased supportive supervision visits from biweekly to weekly in the first month of implementation to provide facilitators with additional coaching.

### Scale-Up Adaptations

The RF approach informed needed adaptation for scale-up, including task shifting from CBO staff to *relais communautaires* (community health workers) as facilitators of video sessions with parents and community members. This change is intended to support sustainability, as relais communautaires are part of the formal health infrastructure, receive periodic training, and are supervised by the Health Zone Leadership Team. In addition, the scope of their work, which includes community-based sensitization activities, is well suited to the group-based discussion format of the video sessions. Though all partners understood the rationale for task-shifting, it did cause tension with CBO partners, who saw it as a reduction of their role and importance in the project. In the resource-constrained context of Kinshasa slums, CBOs also keenly felt the associated reduction in funding and capacity-building opportunities. Use of relais communautaires instead of CBO staff is being piloted during scale-up (Phase 5).

### Rapid Response to Environmental Changes

Finally, RF was critical to our ability to adapt to the evolving implementation environment in Kinshasa. From the beginning, we had to revise our implementation approach to meet realities on the ground—contrary to expectations, there were no existing school-based platforms for VYA clubs, and fluctuations in enrollment due to extreme mobility of the population in Kinshasa required an unanticipated mapping of all schools in our intervention area. Adaptive strategies made possible by RFMs also permitted agility through political instability surrounding the 2018 election, which caused program delays and ushered in a new administration that brought policy changes, including a law supporting free universal primary education. Program delays were addressed via a more intensive implementation schedule (i.e., twice weekly VYA club meetings) similar to the approach used during the learning lab. This permitted realignment to the initial project timeline. Changes to education policy required a shift in our implementation strategy midproject to focus more on in-school implementation and institutionalization. Local partners conducted a mapping exercise to track formerly out-of-school children as they enrolled in school and to integrate them into in-school activities where possible. Community clubs were also reformulated and, in some cases, combined to ensure adequate numbers. This shift prompted an increased focus on institutionalizing in-school components of GUG to reach VYAs, given the very small number of adolescents who were not enrolled in school. Finally, RF approaches enabled a rapid response to disruptions due to school closures during the COVID-19 pandemic, including a shift to distance learning platforms on radio and television.

RFMs help the project team adapt to the evolving implementation environment in Kinshasa, where—from the beginning—we had to adjust our approach to meet on-the-ground realities.

## KEY LEARNINGS IN USE OF RESPONSIVE FEEDBACK

This case study from an ASRH program in Kinshasa provides important learning about how RF can be established and cultivated for the pilot to scale-up life cycle of a community-based health program. We found that creating a culture of learning that embraces and learns from failure starts with staff who model this approach. Our core team internalized this culture and created open, transparent, and 2-way communication with local partners and stakeholders. This process required both an initial investment and continued support.

Initial investment centered on establishing strong processes and structures for meeting regularly to examine and apply learning. Identifying and engaging key stakeholders—including implementing partners, parents, teachers/school staff, and adolescents via the Youth Advisory Council—to participate in these processes was also important. Implementing partners were unaccustomed to playing such a central and trusted role informing program evolution and required coaching and encouragement following initial training and learning meetings. We used some of the same critical reflection and discussion techniques used in the intervention to encourage sharing and spent time reviewing actions that resulted from partner observations so they could see how their input was being applied practically. We also used their feedback to improve RF tools and systems (like monitoring processes) along the way. This commitment to including implementer perspectives and participation in RF was not only an important opportunity for capacity-building but also motivated partner engagement and ownership.

Our commitment to including implementer perspectives and participation in RF was not only an important opportunity for capacity-building but also motivated partner engagement and ownership.

Each RFM had different advantages and challenges. Application of more formal research, such as the outcome evaluation results, to improve program implementation was difficult due to the length of time research firms needed to collect, analyze, and share the data. Similarly, although the learning studies were meant to be rapid to provide real-time data, in practice, the time from conceptualizing the study to sharing the results for application was too slow to be maximally useful. Nevertheless, the GUG project team prioritized sharing results from the outcome evaluation, Global Early Adolescent Study cohort survey, and learning studies among stakeholders. This provided additional opportunities to engage in dialogue with stakeholders and provided important confirmation of issues emerging from monitoring and practice-based data sources. Confirmation with the research study data was critical because, although the monitoring data were available almost immediately to stakeholders, challenges with initial data collection and manual compilation in some cases resulted in data quality issues, leaving questions about the monitoring data's reliability. One example of this was data on exposure to the intervention, which was reported as very low by the outcome evaluation. By contrast, self-reported monitoring data from CBOs showed high and consistent participation throughout the intervention; a subsequent data quality audit confirmed that evaluation measures were likely accurate. In addition, implementer observations were readily available and the most detailed source of information, but they were not always generalizable to all intervention zones.

Finally, RF itself must remain flexible and change over the program cycle. Certain aspects of our RF approach, such as learning meetings and our commitment to collecting and triangulating multiple sources of data, were consistent throughout all phases of program implementation, but others changed along with program needs. Monitoring tools such as the quality benchmarks were added to existing tools as a need arose for more detailed data on facilitation quality, and learning tools changed as well. We traded our simple learning matrix for the more systematic IMT as we entered the scale-up phase. Monitoring tools and indicators were also simplified for scale-up and a new set of benchmarks developed to assess scale-up progress.

In the future, we would advise improving monitoring by tracking participation of youth and their parents in different program components to allow linked, real-time analysis of intervention depth and coverage. This would require a unique code for each child-parent dyad and a tracking system such as stickers, bar codes, or a participation card. This information would enable analysis of the effect of intervention fidelity on outcomes. In addition, given the issues with the quality of monitoring data and the burden collecting this data puts on program staff, we recommend adopting automated approaches for rapidly gathering, collating, and presenting monitoring data, such as using tablets or digital approaches through interactive voice response programs or smartphones (resources permitting). Finally, to understand and improve multilevel norms-shifting interventions, such as GUG, monitoring should be strengthened at all levels of the socioecological system implicated in the program's TOC. In the case of GUG, that would mean gathering monitoring data from caregivers and children and tracking the diffusion of new ideas to the broader community.

We hasten to note that donor support and relationships were critical in allowing us to pursue our RF approach. GUG was supported by 2 donors equally committed to the program vision, who worked collaboratively to provide technical and financial resources. They placed a high amount of trust in our ability to document learning and implement adaptations for increased impact and provided us with latitude to experiment, fail, and learn to improve. Their commitment to RF and adaptive programming were vital to our success.

Programmatic adjustments that resulted from RF are documented in the GUG Implementation Guide—a step-by-step resource for organizations that wish to adopt the intervention.^18^ It provides users with guidance; tested tools; and materials for planning, implementing, supervising, and monitoring this successful norms-shifting intervention.

## CONCLUSIONS

The GUG team of implementers, researchers, donors, and stakeholders realized that success was more likely if the team developed a learning culture, worked closely with stakeholders to develop consensus on a TOC, and gathered and applied information to implementation decisions. RF resulted in an environment where stakeholders had access to the data, skills, and processes to determine whether GUG met scalability criteria, as well as provide critical insight into how to improve the approach for scale. The GUG team collected, analyzed, interpreted, and applied multiple sources of information. This process of triangulation was fundamental to decision-making. Many implementation challenges were first identified by implementers. Their early feedback was almost always confirmed by other sources of data, such as monitoring data or rapid study results. In several instances, we would not have understood the full context of the quantitative data without the accompanying qualitative or “soft” data (e.g., anecdotes). The ability to confirm emerging learnings from multiple perspectives and data sources made it easier to make informed programmatic decisions.

As this case study demonstrates, RF approaches are vital to norms-shifting programs such as GUG, which seek to catalyze change within complex, dynamic systems. In the GUG experience, RF approaches were critical to actively monitor and adjust norms-shifting program processes and effects without delay. We paid close attention to how new ideas and information spread or diffused through communities, what actions and reactions were occurring within communities, how change happened, and which actors were involved. As compared to traditional research efforts, these RFMs supported a more immediate identification of and responses to both the expected and unexpected consequences of program activities. These systematic and timely course corrections ensured programmatic feasibility and acceptability in Kinshasa and helped foster policy support for scale-up efforts and project sustainability. The lessons learned from this project can be extended to other country settings.

## Notes

**Peer Reviewed**

**First published online:** May 10, 2023.
